# Antioxidant Properties of Sourdoughs Made with Whole Grain Flours of Hull-Less Barley or Conventional and Pigmented Wheat and by Selected Lactobacilli Strains

**DOI:** 10.3390/foods9050640

**Published:** 2020-05-15

**Authors:** Viola Galli, Manuel Venturi, Simona Guerrini, Massimo Blandino, Simone Luti, Luigia Pazzagli, Lisa Granchi

**Affiliations:** 1 Department of Agriculture, Food, Environment and Forestry (DAGRI), University of Florence, Piazzale delle Cascine 18, 50144 Florence, Italy; viola.galli@unifi.it (V.G.); lisa.granchi@unifi.it (L.G.); 2 FoodMicroTeam s.r.l., Via di Santo Spirito, 14, 50125 Florence, Italy; simona@foodmicroteam.it; 3 Department of Agricultural, Forest and Food Sciences, University of Turin, Largo Braccini 2, 10095 Grugliasco (TO), Italy; massimo.blandino@unito.it; 4 Department of Biomedical, Experimental and Clinical Sciences “Mario Serio”, University of Florence, Viale Morgagni 50, 50134 Firenze, Italy; simone.luti@unifi.it (S.L.); luigia.pazzagli@unifi.it (L.P.)

**Keywords:** sourdough, lactic acid bacteria, antioxidant activity, whole grain flours, barley flour, pigmented wheat, ex vivo assay

## Abstract

The use of sourdough fermentation and whole grain flours in baked goods manufacturing are known to enhance their functional and nutritional features. In this context, it is necessary to select the most suitable lactic acid bacteria strains and flour combination to achieve this goal. A characterization of 70 lactobacilli strains based on pro-technological and nutritional properties was carried out. The screening allowed the selection of 10 strains that were used to ferment sourdoughs made with two varieties of common wheat, the conventional red-grained cv Aubusson, a blue-grained variety rich in anthocyanins cv Skorpion, and a hull-less barley variety, cv Rondo. From each fermented sourdough, a water soluble extract was obtained and evaluated for its antioxidant activity performed on cultured cells (RAW 264.7 murine macrophage) by assaying Reactive Oxygen Species (ROS) content. Sourdoughs made with pigmented wheat and barley, had an antioxidant activity greater than that recovered in those made with conventional wheat flour, in spite they have been inoculated with the same LAB strains. Results highlighted the interdependence between flour and the inoculated lactic acid bacteria that has to be taken into account for the development of healthy breads exploiting high functional value cereals through biotechnological processes.

## 1. Introduction

An increased demand of consumers for healthy foods has led to the development of breads with whole grain flours, minor and ancient cereals, pseudocereals, legumes, etc. as a strategy to obtain functional products [1]. The consumption of whole grain flour has been demonstrated to reduce cardiovascular disease, diabetes, obesity, and colorectal cancer [2,3,4,5]. These beneficial effects are due to the high content of dietary fiber such as non-starch polysaccharides occurring in whole grain flour, even if other components including phenolic compounds may be involved [2]. In particular, some pigmented whole grains are innovative and valuable raw materials for the production of functional foods. Together with phenolic acids these grains show significant levels of anthocyanins and carotenoids, bioactive compounds responsible for their characteristic blue-purple and yellow-orange colors, and for their increase nutritional value [6,7,8,9]. Some authors have reported their use for enhancing the nutritional quality of baked products such as biscuits [10], bread [11,12] and pasta [13]. Whole grain and particularly pigmented cereals are, hence, an important source of bioactive compounds, micronutrients, and dietary fiber; however, if the outer layers of the seed are separated from the endosperm, several bioactive compounds are lost [14]. Another strategy to enhance the nutritional quality of bread is to increase the content of β-glucan. Cereal β-glucans are able to lower postprandial serum glucose levels and insulin response and to reduce cholesterol levels [15]. The higher concentration of β-glucan in hull-less barley than in wheat has made this cereal interesting for bread production. Nevertheless, despite the nutritional value of the whole grain flours, the incorporation of significant percentages of whole grain wheat or barley flour in the dough reduces the technological quality of bread, especially the loaf volume. This problem is due to the dilution of wheat gluten and to the soluble and insoluble fibers, that, binding water, mechanically interfere with gluten network formation [16,17], thus affecting the gas-holding capacity of doughs. One approach to improve bread quality, without using additives, is to employ the sourdough technology, thanks to the multiple metabolic activities of lactic acid bacteria (LAB) that carry out the dough fermentation together with yeasts. Sourdough fermentation represents an option for the whole grain products manufacturing and can be used to improve whole grain flours functionality [18]. However, this technology is difficult to standardize, hence this aspect has to be taken into particular consideration when the aim is to produce bread with specific nutritional and/or nutraceutical characteristics. Therefore, to get optimal technological, sensory and healthy properties, is of key importance setting-up a proper biotechnology process [19]. One suitable tool to get this goal is the use of microbial starters able to guarantee fast, efficient, controllable, and reproducible fermentations [20]. The main criteria used to select LAB starter are technological, sensory, and nutritional properties. Technological factors of interest for rapid sourdough fermentations are microbial growth and acidification rate [21], whereas the nutritional properties are the capability to degrade anti-nutritional factors, to increase the availability of functional compounds [22,23], and to produce bioactive compounds, such as peptides, and amino acid derivatives with various functionalities. The LAB selection includes the study of a large number of strains for different metabolic activities, which should be also confirmed in the type of flour used for the bread production. Indeed, flour is one of the main parameters contributing to the selection of a stable microbiota in sourdough [24] and conferring consumer health benefits because of its content in bioactive compounds such as phenolic acids, anthocyanins, and carotenoids. Therefore, the aim of this study was to define potential bacterial starters to produce functional whole grains bread by using whole grain wheat and barley flours. Initially, different LAB strain/flour combinations were assayed for their pro-technological capabilities; then, only the best performing combinations were tested for their antioxidant activities on cell cultures (RAW 264.7 murine macrophages) in ex vivo assays. The LAB strains used in this study were 70 and were isolated from different Italian sourdoughs, while the whole grain flours were of a conventional (Aubusson) and a pigmented (Skorpion, with blue grains) cultivar of common wheat and one of hull-less barley (Rondo).

## 2. Materials and Methods

### 2.1. Grain Cultivation and Milling

In this study, three different whole grain flours were analysed. In particular, two varieties of ordinary bread-making winter common wheat (*Triticum aestivum* spp. *aestivum* L.), a conventional red-grained variety (cv Aubusson, Limagrain Italia SpA) and a blue-grained variety rich in anthocyanins (cv Skorpion, Agricultural Research Institute Kromeriz, Ltd., Kroměříž, Czech Republic) were used. In addition a hull-less spring barley variety (*Hordeum vulgare* L. *var. nudum* Hook, cv Rondo, Società Italiana Sementi s.p.a., San Lazzaro di Savena, Italy) was considered. All previous reported cultivars were grown side by side on the same experimental field located in Carmagnola Italy (Piedmont; 44°50′ N, 7°40′ E; altitude 245 m) during the growing season 2016/2017. The plot size for each cultivar was 5 × 100 m (500 m^2^) and the ordinary crop technique of the growing area was applied. Whole grain flour (20 kg) from each cultivar was obtained by means of a single-stream natural stone milling process (Molino Tomatis S.N.C, Niella Tanaro, Italy). The whole grain milling was carried out without any sifting processes, thus all anatomical kernel components, including the endosperm, germ, and bran are present in the same relative proportions as in the intact kernel. On average, the particle-size distribution of the whole grain flours was 15%, 50% and 35% for fine ( <250 µm), medium (250–710 µm) and coarse ( >710 µm) grinding fractions After each operation, the mill was cleaned thoroughly by aspiration to avoid equipment contamination and washed with alcohol to minimize microbial contamination.

### 2.2. Functional Characterization of Flours

The whole grain flour from each cultivar was analyzed for soluble and cell wall-bound phenolic acids, total anthocyanins and total antioxidant capacity.

#### 2.2.1. Extraction of the Soluble (SPAs) and Cell Wall-Bound Phenolic Acids (CWBPAs) and Quantification by Means of RP-HPLC/DAD

The extraction of soluble (free and conjugated) and cell wall-bound phenolic acids was performed according to Li et al. [25] with some modifications. DHB was used as internal standard to ensure that losses due to the extraction method were accounted for. One hundred and twenty-five milligrams of each sample were mixed with in 1 mL of 80:20 (*v*/*v*) ethanol: water solution. The resulting mixtures were vortexed for 30 s, and then sonicated (35 kHz, Sonorex Super RK 156 BH, Bandelin Electronic, Berlin, Germany). Samples were centrifuged at 10,600× *g* for 10 min (Centrifuge 5417R, Eppendorf, Hamburg, Germany), the supernatants were removed, and a second extraction was carried out with 1 mL 80:20 (*v*/*v*) ethanol: water solution. For the extraction of soluble and cell wall-bound phenolic acids, the supernatants and the pellets were collected, respectively. The combined supernatants collected in the first part of the extraction were completely evaporated (Sample Concentrator FSC496D, Techne, UK) under a nitrogen stream and the dry residue was hydrolyzed with 2 M NaOH (400 μL) for 2 h under continuous agitation at 4 °C. After acidification with HCl, soluble phenolic acids were extracted with 500 μL of ethyl acetate. After centrifugation at 10,600× *g* for 2 min, the upper layer was transferred in a clean microcentrifuge tube. The extraction was repeated twice, and the ethyl acetate extracts containing soluble phenolic acids were combined in the same microcentrifuge tube.

All the amount of pellet remaining after the removing of supernatants in the first part of the extraction was hydrolyzed 4 h under continuous agitation at 4 °C, by adding 2 M NaOH (400 μL). After acidification with HCl, the bound phenolic acids were extracted with 800 μL of ethyl acetate and then centrifuged at 10,600× *g* for 2 min. The extraction was repeated another time and the ethyl acetate extracts containing cell wall-bound phenolic acids were combined in the same microcentrifuge tube.

Ethyl acetate was evaporated to dryness under a nitrogen stream, and then each sample was reconstituted in a 80:20 (*v*/*v*) methanol: water solution. Phenolic extracts were filtered through a 0.2 μm filter and then analyzed by means of a high-performance liquid chromatograph Agilent 1200 Series (Agilent Technologies, Santa Clara, CA, USA) coupled to an Agilent 1200 Series diode array detector. Separations were carried out using a 150 × 4.6 mm, 5 μm, Gemini RP-18 column (Phenomenex, Torrance, CA, USA); the column temperature was set at 35 °C. The mobile phase consisted of 0.1% acetic acid in water (solvent A) and 0.1% acetic acid in methanol (solvent B). The following operating linear gradient was used: 0–22 min, 9–42% B; 22–27 min, 42–90% B; 27–32 min, 90% B. Finally, the mobile phase was brought to 9% B in 3 min, and this was followed by 16 min of equilibration. The flow rate of the mobile phase was 1 mL/min. Phenolic acids were identified using the retention times and the UV/Vis spectra of their respective standards. Individual phenolic acid standards were also prepared and diluted to different concentrations to obtain calibration curves for quantification purposes.

#### 2.2.2. Extraction and Quantification of Total Anthocyanins

The total anthocyanin content (TAC) was determined only for the stone-milled flour obtained from the blue-variety of common wheat (cv Skorpion), since these compounds were absent in conventional red-grained cultivars [26]. Each sample (1 g) was extracted using 8 mL of ethanol acidified with HCl 1 N (85:15, *v*/*v*) for 30 min. The absorbance was measured after centrifugation at 20,800× *g* for 2 min at 540 nm, as reported by Siebenhandl et al. [6]. The total anthocyanin content was expressed as mg cyanidin-3-*O*-glucoside (Cy-3-glc) equivalents/Kg of sample (dw).

#### 2.2.3. Determination of Antioxidant Capacity (AC) by Means of the FRAP Assay

Tre FRAP assay adapted into QUENCHER method was performed as described by Serpen et al. [27]. Briefly, FRAP reagent was prepared by mixing a aqueous solution of 10 mM TPTZ and 20 mM ferric chloride in 300 mM sodium acetate buffer (pH 3.6) at a ratio of 1:1:10 (*v*:*v*:*v*). Samples (2 mg) were analyzed by adding FRAP working solution (2 mL). The reaction was carried out under stirring at 1000 rpm (PCMT Thermoshaker, Grant Instruments, Cambridge, UK). After 120 min, samples were centrifuged for 1 min at 20,800× *g*, and the absorbance of the supernatant was measured at 593 nm (Cary 60 UV-Vis, Agilent Technologies). The final results were expressed as mmol Trolox equivalents/kg of sample (dw).

#### 2.2.4. Determination of Antioxidant Capacity by Means of the ABTS Assay

The ABTS assay adapted into QUENCHER method was performed as reported by Serpen et al. [27]. Briefly ABTS + working solution was obtained by diluting the aqueous stock solution (7 mM ABTS, 2.45 mM potassium persulfate) with water until the absorbance at 734 nm was 1.5. Samples (1.8 mg) were analyzed by adding 900 μL of ethanol and 900 μL of ABTS working solution (final solvent ratio-water: ethanol 50:50). The reaction was carried out under stirring at 1000 rpm. After 30 min, samples were centrifuged for 1 min at 20,800× *g*, and the absorbance of the supernatant was measured at 734 nm. The final results were expressed as mmol Trolox equivalents/Kg of sample (dw).

### 2.3. Microorganisms and Culture Conditions

70 lactobacilli (LAB) strains belonging to the collection of Department of Agriculture, Food, Environment and Forestry (DAGRI) of the University of Florence (Italy), were used. The LAB strains belonged to five *Lactobacillus* species (*L. brevis*, *L. farciminis*, *L. plantarum*, *L. rossiae,* and *L. sanfranciscensis*), as reported in Table 1, and were isolated from Italian wheat sourdoughs used to produce various baked goods such as Colomba, Panettone, Lagaccio, and breads. Before assaying, they were routinely propagated for 24 h at 30 °C in MR3i [23], a medium containing (in g/L): maltose 20, glucose 6, fructose 6, polypeptone 10, meat extract 5, yeast extract 12, sodium gluconate 2, sodium acetate trihydrate 5, ammonium citrate trihydrate 2, di-potassium hydrogen phosphate 2, magnesium sulfate heptahydrate 0.2, manganese sulfate tetrahydrate 0.05, cysteine-HCl 0.5, vitamin mix 1 mL, Tween 80 1 mL, fresh yeast extract 15 mL, pH of 5.6.

### 2.4. Proteolytic Activity of Lactic Acid Bacteria

LAB isolates were grown overnight at 30 °C in the MR3i in incubator (Vismara, Sant’Angelo Lodigiano, Lodi, Italy) medium before carrying out the assays. Bacterial cell counts were estimated using a Neubauer improved counting chamber (Marienfeld, Lauda-Königshofe, Germany) in order to inoculate 10^9^ cell/mL into Gluten Maltose Broth (GMB), a medium containing gluten as sole nitrogen source as described by Pepe et al. [28]. At the beginning and after 24 h, GMB cultures were centrifuged at 10,000× *g* for 10 min and supernatants were used to determine hydrolyzed gluten proteins using Bradford spectrophotometric assay (Sigma Aldrich, St. Louis, MO, USA). A calibration curve was prepared using BSA (bovine serum albumin) (Sigma Aldrich) as standard, at concentrations ranging from 0 to 200 mg/L. The absorbance increase was measured using un-inoculated GMB as control and proteolytic activity of each LAB culture was expressed as BSA mg/L.

### 2.5. Peptidase Activity of Lactic Acid Bacteria

Stationary phase cells grown overnight in MR3i broth and counted by a Neubauer improved counting chamber (Marienfeld, Lauda-Königshofe, Germany) to obtain a cell suspension of 10^9^ cell/mL concentration, were harvested by centrifugation at 12,000× *g* for 5 min, washed twice with sterile 50 mM potassium phosphate buffer, pH 7.0, and re-suspended in the same buffer. Two general aminopeptidase (PepNC), proline iminopeptidase (PepI), glutamyl aminopeptidase (PepA) and X-prolyl dipeptidyl aminopeptidase (PepX) activities were measured according to Macedo et al. [29] using Lys-*p*-nitroanilide (pNA), Leu-p-NA, Pro-p-NA, Glu-p-NA and Gly-Pro-p-NA (Sigma Aldrich,) respectively, as synthetic substrates. The assay mixture contained 30 μL of a 20 mM, synthetic substrate methanol solution; 195 μL of 50 mM potassium phosphate buffer, pH 7.0; 95 μL of 0.05% (*w*/*v*) sodium azide solution; and 75 μL of cell suspension. After incubation at 30 °C for 4 h, the reaction was stopped by addition of 900 μL of 10% (*v*/*v*) acetic acid. The release of *p*-nitroanilide (*p*-NA) was measured spectrophotometrically at 410 nm after centrifugation of the reaction mixture at 12,000 × *g* for 5 min. To determine the peptidase activity, the data obtained were compared to a calibration curve prepared using p-NA at concentrations in the range 0.1–20.0 mM.

### 2.6. Sourdough Fermentation and Water-Soluble Extracts

LAB strains were grown overnight in MR3i broth at 30° in the incubator (Vismara, Sant’Angelo Lodigiano, Lodi, Italy). For the inoculum in sourdoughs, cells were recovered by centrifugation (5000× *g* for 20 min), successively washed in physiological solution and re-suspended in the tap water used for the preparation of the doughs, in order to inoculate a concentration of ca 10^8^ CFU/g. According to the commonly used backslopping conditions, the doughs were prepared by mixing water and flours of each cultivar to obtain a dough yield [DY = (mass of dough/mass of flour) × 100] of 160 and were fermented for 24 h at 30 °C in the incubator (Vismara). The water-soluble extracts (WSE) of dough samples were obtained by extracting doughs, after 8 h of fermentation, with sterile water (1:3 *w*/*v*) and centrifugation at 14,000× *g* for 20 min at 4 °C according to Galli et al. [30].

### 2.7. Enumeration of Lactic Acid Bacteria

10 g of sourdough sample, transferred into 90 mL of sterile physiological solution, were homogenized for 2 min in a Stomacher Lab Blender 400 (Seward Ltd., Worthing, West Sussex, UK). After decimal dilutions, 100 µL of these suspensions were plated on MR3i agar for cell enumeration. Plates were placed in jars with the enzymatic kit AnaeroGen (Oxoid Ltd., Hampshire, UK) for obtaining anaerobic conditions to simulate dough microaerophilic condition and LAB counted after 48–72 h at 30 °C. Plate counts were performed in duplicate.

### 2.8. Determination of pH and Total Titratable Acidity

The pH values were determined by a pH-meter (Metrohm Italiana Srl, Varese, Italy) every hour until 8 h and successively after 24 h of fermentation (only for the screening of the 70 lactobacilli). Total titratable acidity (TTA) was measured on 10 g of dough samples, which were homogenized with 90 mL of distilled water for 3 min. TTA was expressed as the amount (mL) of 0.1N NaOH necessary to achieve a pH of 8.5 after 8 and 24 h (only for the screening of the 70 lactobacilli). The analyses were carried out in duplicate.

### 2.9. Ex Vivo Assays

Water soluble extracts from sourdoughs were assayed for protein and peptide content by the bicinchoninic acid method (BCA, Pierce Chemical, Rockford, IL, USA). RAW 264.7 murine macrophages (Sigma Aldrich) were cultured in standard conditions (5% CO_2_, 37 °C) in Dulbecco’s modified Eagle medium (DMEM) and then added with 10% (*w*/*v*) foetal bovine serum (FBS), 100 µg/mL penicillin/streptomycin and 1 mM glutamine. The cells were routinely sub-cultured every two days. To determine antioxidant capacity, cells were put in 96-well at 2 × 10^5^ cells/well; they were treated for 1 h with LMW extracts at final concentration of 0.05 mg/mL and then co-incubated with 1 µg/mL of lipopolysaccharide (LPS) for 24 h. Stressed cells were used as positive control, while cells treated with 0.05 mM of ascorbic acid were used as negative control. The level of intracellular Reactive Oxygen Species (ROS) was assessed by measuring the oxidation of the probe 2′,7′–dichlorofluorescin diacetate (25 μM DCFH-DA in DMSO). The probe was added to the medium and the cells were incubated for 1 h. The cells were lysed with the RIPA buffer (50 mM TRIS-HCL, 150 mM NaCl, 100mM NaF, 2 mM EGTA, 1% Triton x-100) and then centrifuged at 10,000× *g* for 10 min. A Fluoroscan Ascent FL microplate fluorescent reader (Thermo Electronic Corporation, Waltham, MA, USA) was used to detect fluorescence at the excitation/emission wavelengths of 485/538 nm. The values were determined as fluorescence intensity units and the results expressed as a percentage of the reduction in ROS formation.

### 2.10. Statistical Analysis

The numerical results of microbial, and chemical, analysis in this study are averages of two or three independent replicates. Data were analyzed by one-way analysis of variance (ANOVA) using GraphPAD Prism 6. The means comparisons were determined by Tukey’s test (*p* < 0.05). Kinetics of microbial growth and acidification were modelled according to the Gompertz equation as modified by Zwietering et al. [31] using GraphPAD Prism 6 (GraphPad Software, San Diego, CA, USA).

## 3. Results

### 3.1. Flour Functional Characterization

In order to evaluate some functional properties of the flour used to make sourdough and, hence, to select LAB strains, phenolic acids as well as the antioxidant activity of the three different whole grain flours (winter wheat cv Aubusson and cv Skorpion and spring barley cv Rondo) were determined. As expected, independently of the flour variety, CWBPAs contributed the highest proportion of the total phenolic acids (Table 2). However, the content of these compounds in the hulls-less barley variety Rondo and in blue-grained wheat variety Skorpion were significantly higher than the content that occurred in the red-grained wheat flour variety Aubusson. The SPAs showed different values according to the flour variety with the cv Rondo containing the lowest concentration. The antioxidant capacity, measured by two methods, resulted significantly higher in barley flour than in both the wheat flours. Flour from the blue-variety of common wheat cv Skorpion contained concentrations of total anthocyanin, comparable to values found in other blue wheat variety [32].

### 3.2. LAB Proteolytic and Peptidase Activities

**Figure 1:**
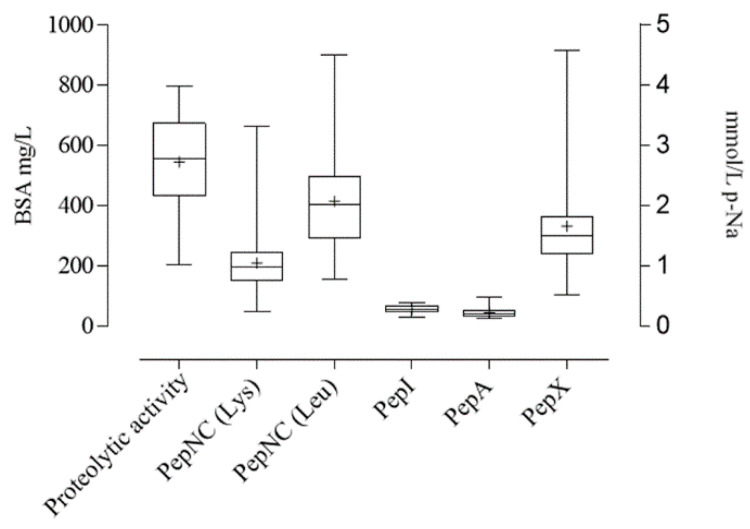
Box plot representing LAB proteolytic activity (expressed as bovine serum albumin (BSA) mg/L and plotted on the left y-axis) and peptidase activities (expressed as *p*-nitroanilide (p-Na) mmol/L and plotted on the right axis). General aminopeptidase (PepNC) on L-lysine-*p*-nitroanilide and L-leucine-*p*-nitroanilide, proline iminopeptidase (PepI) on L-proline-*p*-nitroanilide, glutamyl aminopeptidase (PepA) on L-glutamic acid-*p-*nitroanilide and X-prolyldipeptidyl aminopeptidase (Pep X) on Gly-Pro-*p*-nitroanilide. The center line of each box represents the median, the mean is indicated by “+”, the top and bottom of the box represent the 75th and 25th percentile, respectively.

The 70 lactobacilli strains were tested for their proteolytic and peptidase activities. The results showed a high variability among the strains (Figure 1). The proteolytic activity of the strains ranged from 796 to 205 mg BSA/L, with a median value of 555 mg BSA/L. This variability did not depend on the LAB species nor on their origin of isolation. Proline iminopeptidase (PepI) and glutamyl aminopeptidase (PepA) activities were lower compared to the other enzymatic activities. The median values of pNa released were 0.27 and 0.19 mM for PepI and PepA respectively, ranging from 0.13 to 0.47. PepNC ranged from 0.23 to 4.50 mM of pNa released, with a median value of 0.98 and 2.00 for lysine aminopeptidase and leucine aminopeptidase, respectively. PepX activities range was between 0.52 and 4.57 mM of pNa released.

### 3.3. LAB Pro-Technological Properties

**Figure 2:**
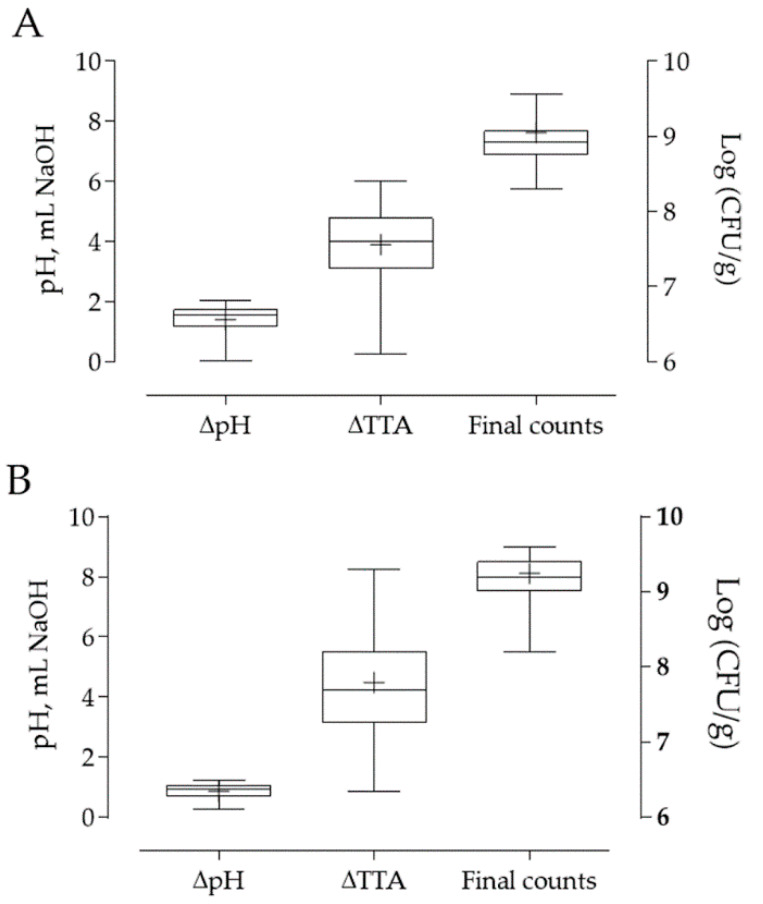
Box plot representing pH variations (∆pH), TTA variations (∆TTA) and microbial concentrations determined after 8 h of fermentations in sourdough obtained with wheat (**A**) or barley (**B**) flour and inoculated with 70 different LAB strains. The centre line of each box represents the median, the mean is indicated by “+”, the top and bottom of the box represent the 75th and 25th percentile, respectively.

The lactobacilli strains, were at first screened for growth and acidification capabilities in sourdoughs obtained with each cereal flours, in order to investigate their pro-technological features. Since no differences were observed between the two wheat varieties, results of cv Skorpion were not reported. The results of pH variations (∆pH), TTA variations (∆TTA) and microbial concentrations quantified after 8 h of fermentations are shown in Figure 2 as box and whiskers graphs for each flour. Acidification data determined after 24 h of fermentation levelled the differences among the strains, hence were not considered for the selection.

The ∆pH was greater in wheat than in barley flour, while the ∆TTA was not significantly different in the two flours (*t*-test, *p* < 0.05). Finally, the growth capability of the LAB was significantly higher in barley flour than in wheat flour (*t*-test, *p* < 0.05). Considering the data obtained for each strain, the variability of the acidification capacity observed among the tested LAB strains was similar in the two flours (variation coefficients of about 30 and 35% for ∆pH and ∆TTA respectively). The differences among strains were more evident in the final counts, showing variation coefficient of 70 and 50% for barley and wheat, respectively.

### 3.4. Selection of LAB Strain/Flour Combinations

LAB strains were grouped in four classes based on the percentile distribution of the values of proteolytic and peptidase activities, ∆pH, ∆TTA, and microbial concentrations determined after 8 h of fermentation. 10 strains showing a greater number of parameters in the 75th percentile were selected. Lactobacilli that demonstrated the best performance on wheat flour were two *L. farciminis* strains (Fi14 and Fi17), five *L. plantarum* strains (Fi8, Fi13, Fi15, Fi27 and Fi58), two *L. rossiae* (Fi40 and Fi19), and one *L. sanfranciscensis* (Fi18). Similarly, the best performing LAB strains on barley flour were two *L. farciminis* strains (Fi32 and Fi70), three *L. plantarum* strains (Fi15, Fi31 and Fi58), three *L. rossiae* (Fi19, Fi40 and Fi21), one *L. sanfranciscensis* (Fi33), and one *L. brevis* (Fi30). Only four strains were in common to the two flours: *L. plantarum* Fi15, *L. plantarum* Fi58, *L. rossiae* Fi19, and *L. rossiae* Fi40.

### 3.5. LAB Acidification Kinetics

The strain/flour combinations previously selected (20 in total) were assayed for their fermentative performance in terms of maximum pH variation at the end of fermentations (∆pH), maximum specific pH decrease rate (μ max), and length of lag phase using Gompertz-model. In Table 3 and Table 4 the acidification parameters of the strains assayed on wheat and barley flour are reported.

The goodness of fit of the Gompertz model was appropriate for all the strains assayed, *R*^2^ values being higher than 0.95 (data not shown). The 10 lactobacilli strain/wheat flour combinations showed no significant difference in ∆pH and µ max (ANOVA; *p* < 0.05) with mean values around 2.2 pH unit and 0.4 ∆pHh-1, respectively. On the contrary, the lag phase was shorter for some bacterial strains, ranging from 1 h (*L. rossiae* Fi40) to 3 h (*L. plantarum* Fi13). No statistically significant difference (ANOVA; *p* > 0.05) was found between the acidification parameters in the strain/barley flour combinations. The values of ∆pH, µ max and lag phase were about 1.2 pH unit, 0.4 ∆pHh^−1^ and 3 h, respectively.

### 3.6. Intracellular Reactive Oxygen Species (ROS) Assay

The best performing LAB strain/flour combinations were used to carry out sourdough fermentation prepared with the three different whole grain flours. In particular, the same combinations were employed in sourdoughs made with the two wheat varieties, cv Aubusson and cv Skorpion, the latter being rich in anthocyanins.

**Figure 3:**
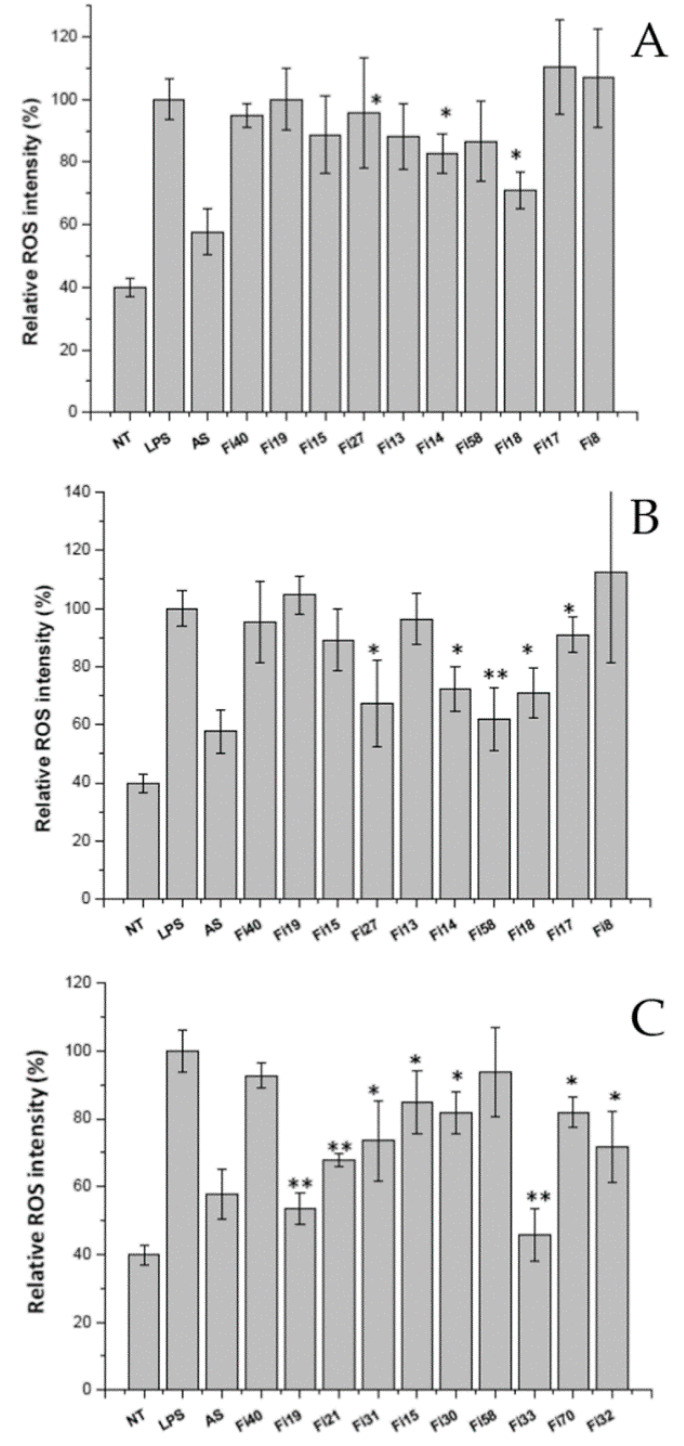
Intracellular ROS levels in RAW cells treated with water soluble extracts obtained from sourdough inoculated with different lactic acid bacteria and prepared with wheat flour cv Aubusson (**A**), cv Skorpion (**B**), and barley flour cv Rondo (**C**). Cells were treated with 0.05 mg/mL water-soluble extracts and 1 µg/mL LPS as reported in methods. NT: Not Treated cells; LPS: positive control, cells treated with the only LPS; AS, ascorbic acid, cells treated with ascorbic acid and LPS. Results are the mean (±SD) of two different experiments performed in duplicate. Statistical analysis was performed for each sample vs the LPS value (** *p* < 0.001; * *p* < 0.05).

At the end of 8 h of fermentation, the water salt soluble extracts of sourdoughs prepared with wheat and barley flours and inoculated with the selected 10 strains were obtained and tested to evaluate their possible antioxidant effect on cell cultures in ex vivo assays. The extracts were assayed for protein concentration (data not shown) and then they were lyophilized and dissolved in bi-distilled water to obtain samples with the same protein concentration that were used to treat cells. LPS was used as stressing molecule to induce reactive oxygen species (ROS) synthesis. ROS were determined by co-incubating RAW cells with LPS and water soluble extracts and, consequently, the effect of the extracts was measured as the ability to reverse the ROS production induced by LPS itself in stressed cells. The negative control was performed by treating stressed cells with ascorbic acid, a typical ROS scavenger. The results showed (Figure 3) that sourdoughs obtained with different strains have different antioxidant activities. Only two LAB strains (*L. rossiae* Fi40 and *L. plantarum* Fi8) had no effects on the production of active molecules in none of the sourdoughs. Conversely, other strains, such as *L. farciminis* Fi14, *L. plantarum* Fi58, *L. sanfranciscensis* Fi18, *L. rossiae* Fi19, and *L. sanfranciscensis* Fi33 showed a strong antioxidant activity (*p* < 0.05 for extracts vs LPS value). However, it is worth to notice that reduction of ROS depended both on LAB strain and on flour. In fact, a comparison between Aubusson and Skorpion sourdoughs highlighted that Skorpion sourdoughs had an antioxidant activity greater than that recovered in Aubusson ones, in spite they have been inoculated with the same LAB strains. In addition, most of the sourdoughs obtained with barley flour possessed a strong antioxidant activity; only two samples, fermented with *L. rossiae* Fi40 and *L. plantarum* Fi58 strains, did not show the ability to reduce significantly the ROS production.

## 4. Discussion

Interest in producing healthy bread is growing because of multiple benefits that can be provided to consumers [33,34]. Different strategies can be used to achieve this goal. Considering that the type of flour as well as the bread-making technique may have different effects on consumer’s health, in this work a multistep approach was applied to obtain the most suitable combination starter culture/flour for producing functional baked goods. The considered steps were as follows: (i) characterization of high functional value whole grains flours according to their bioactive compounds and their antioxidant activity (ii) selection of lactobacilli strains based on their technological and functional properties (iii) selection of the best performing combination LAB-strain/flour.

The characterization of whole grains flour included a hull-less barley flour because it provides higher β-glucan content [35] and a blue-grain wheat flour (cv Skorpion) containing high anthocyanin concentrations [26] and they were compared to a conventional red-grained wheat variety (cv Aubusson). Results demonstrated that the total phenolic acids content was higher in wheat flour, variety Skorpion and in barley flour, whereas the highest antioxidant activity was observed in barley flour. These findings are consistent with those reported in other studies [36,37], and underline that that utilization of barley and wheat cultivar rich in bioactive compounds in breads has a beneficial health potential.

The sourdough fermentation was used because lactic acid bacteria, which ferment in consortium with the yeasts, are responsible for reducing dough pH. This condition lowers the glycemic index of bread and promotes the protein and phytic acid degradation, allowing an increase in minerals, free amino acids and protein bioavailability [38]. In addition, other functional/nutritional aspects have been associated to sourdough fermentation in particular thanks to selected LAB strains possessing specific properties. In this work, 70 lactobacilli strains, isolated from sourdoughs and belonging to five species, were selected for their proteolytic and peptidase activities, as recent findings have demonstrated the capability of sourdough lactic acid bacteria to produce bioactive peptides with various antioxidant and anti-inflammatory properties [30,39]. Overall, all lactobacilli strains displayed a high variability in these enzymatic activities, independently of the species, thus resulting strain-specific properties, in agreement with the current literature [40]. Proline iminopeptidase (PepI) and glutamyl aminopeptidase were very low in all tested LAB strains as reported in other papers [30,41]. These microbial activities occurring in sourdough contribute to the degradation of cereal proteins to peptides and amino acids, affecting bread quality as taste-active, flavor precursors or bioactive [42]. The further evaluation was carried out in order to assess the pro-technological features of the lactobacilli. The acidification rate and microbial growth of all 70 LAB strains in sourdoughs prepared with hull-less barley flour (cv Rondo) and wheat flour (cv Aubusson), allowed to select the 10 strains exhibiting the greatest number of parameters in the 75th percentile, in relation to the cereal flour.

No LAB strain belonging to the heterofermentative *L. brevis* species showed, in both flours, appropriate properties to be selected as starter culture despite its frequent occurrence in natural sourdoughs of different origin [43]. On the other hand, different strains belonging to the other four species, *L. plantarum*, *L. rossiae*, *L. farciminis,* and *L. sanfranciscensis*, resulted suitable according to the type of flour. Indeed, it was demonstrated that only four strains, *L. plantarum* Fi15, *L. plantarum* Fi58, *L. rossiae* Fi19, and *L. rossiae* Fi40, were effective in both flour grains whereas other 6 lactobacilli strains were specific according to the cereal flour type. The specific association of LAB strain and flour was also highlighted assaying the antioxidant properties of the different sourdough water soluble extract (WSE) on murine cell cultures. This property is of peculiar interest for the production of breads with improved healthy value, indeed recent findings showed a maintenance of the biological activity of the extracts also after cooking [44]. Our results showed that, for instance, the WSE from the sourdough prepared with Skorpion wheat variety and the strains *L. farciminis* Fi17, *L. plantarum* Fi27, and *L. plantarum* Fi58 displayed significant antioxidant activity whereas the WSE from the sourdough prepared with Aubusson wheat variety and the same lactobacilli strains did not show any activity. Moreover, the strain *L. rossiae* Fi19 exhibited a strong antioxidant activity only when associated to barley flour. In general, a stronger antioxidant activity was found for barley WSE, followed by those obtained from Skorpion sourdough, pointing out the influence of the flour composition on the nutritional features. Indeed, these two whole grain flours showed a higher amount of total phenolic acids than wheat cv Aubusson. Therefore, the most suitable combination of selected LAB and high functional value flour has to be taken carefully into account for producing healthy baked goods by sourdough fermentation. Finally, the use of multiple-strain starter cultures might be suggested, although the interaction among different LAB strains should be investigated.

## Figures and Tables

**Table 1:** Lactic acid bacteria strains isolated from Italian sourdoughs and used in this study.

Species	Strains
*Lactobacillus brevis*	Fi6; Fi7; Fi30; Fi33; Fi35; Fi44; Fi45; Fi49; Fi50; Fi63, Fi67; Fi68
*Lactobacillus farciminis*	Fi1; Fi2; Fi11; Fi14; Fi17; Fi20; Fi29; Fi32; Fi41; Fi42; Fi43; Fi51; Fi70
*Lactobacillus plantarum*	Fi8; Fi13; Fi15; Fi27; Fi31; Fi34; Fi40; Fi52; Fi53; Fi54; Fi55; Fi58; Fi60; Fi61; Fi62
*Lactobacillus rossiae*	Fi4; Fi9; Fi10; Fi12; Fi16; Fi19; Fi21; Fi22; Fi24; Fi25; Fi28; Fi37; Fi38; Fi39; Fi59; Fi69
*Lactobacillus sanfranciscensis*	Fi3; Fi5; Fi18; Fi23; Fi26; Fi36; Fi46; Fi47; Fi48; Fi56; Fi57; Fi64; Fi65; Fi66

**Table 2:** Cell wall-bound phenolic acids (CWBPAs), soluble (free and conjugated forms) phenolic acids (SPAs), total anthocyanin (TAC) and antioxidant capacity (AC, FRAP and ABTS methods) detected in the stone-milled flours of the cereal tested. (Data are expressed on a dw basis ± standard deviation). Means followed by different letters are significantly different, according to the REGW-Q test (the ANOVA level of significance is shown in the table).

Cereal	Cultivar	CWBPAs ^1^	SPAs ^1^	TAC	AC-FRAP	AC-ABTS
		(mg/kg)	(mg/kg)	(mg cya/kg)	(mmol TE/kg)	(mmol TE/kg)
Red-grained wheat	Aubusson	710 ± 29 ^b^	63.6 ± 3.9 ^b^	-	7.5 ± 0.5 ^b^	18.8 ± 0.9 ^b^
Blue-grained wheat	Skorpion	897 ± 19 ^a^	84.7 ± 3.7 ^a^	22.8 ± 0.4	7.9 ± 0.6 ^b^	19.9 ± 0.7 ^b^
Naked barley	Rondo	957 ± 7 ^a^	37.2 ± 3.2 ^c^	-	23.6 ± 1.5 ^a^	31.9 ± 1.8 ^a^
*p* (F)		<0.001	0.001	-	<0.001	<0.001

**Table 3:** Kinetic parameters of lactic acid bacteria acidification, modelled according to the Gompertz equation, of conventional red-grained wheat (cv Aubusson) sourdough fermented at 30 °C for 8 h.

Strain	∆pH (pH Unit)	µ Max (−∆pHh^−1^)	Lag Phase (h)
*L. plantarum* Fi8	2.376 ± 0.326	0.345 ± 0.028	2.224 ± 0.283 ^bc^
*L. plantarum* Fi13	2.172 ± 0.349	0.370 ± 0.029	3.279 ± 0.236 ^c^
*L. plantarum* Fi15	2.309 ± 0.124	0.391 ± 0.019	1.368 ± 0.207 ^ab^
*L. plantarum* Fi27	1.966 ± 0.088	0.434 ± 0.028	1.282 ± 0.205 ^ab^
*L. plantarum* Fi58	1.977 ± 0.121	0.416 ± 0.031	2.227 ± 0.207 ^bc^
*L. farciminis* Fi14	2.232 ± 0.289	0.373 ± 0.028	2.904 ± 0.232 ^c^
*L. farciminis* Fi17	2.337 ± 0.163	0.348 ± 0.016	1.624 ± 0.211 ^ab^
*L. sanfranciscensis* Fi18	1.989 ± 0.095	0.362 ± 0.015	2.279 ± 0.135 ^bc^
*L. rossiae* Fi19	2.208 ± 0.117	0.365 ± 0.017	1.578 ± 0.183 ^ab^
*L. rossiae* Fi40	2.251 ± 0.106	0.349 ± 0.013	0.977 ± 0.197 ^a^

Different letters (a,b,c) indicate significant differences (*p* ≤ 0.05).

**Table 4:** Kinetic parameters of lactic acid bacteria acidification, modelled according to the Gompertz equation, of hull-less barley (cv Rondo) sourdough fermented at 30 °C for 8 h.

Strain	∆pH (pH Unit)	µ Max (−∆pHh^−1^)	Lag Phase (h)
*L. plantarum* Fi15	1.399 ± 0.081	0.310 ± 0.022	2.611 ± 0.178
*L. plantarum* Fi31	1.188 ± 0.180	0.364 ± 0.098	3.456 ± 0.457
*L. plantarum* Fi58	1.266 ± 0.126	0.328 ± 0.068	2.799 ± 0.355
*L. farciminis* Fi32	1.208 ± 0.246	0.381 ± 0.049	2.646 ± 0.355
*L. farciminis* Fi70	1.169 ± 0.150	0.352 ± 0.082	3.284 ± 0.408
*L.sanfranciscensis* Fi33	1.158 ± 0.165	0.373 ± 0.096	3.680 ± 0.411
*L. rossiae* Fi19	1.143 ± 0.134	0.423 ± 0.102	4.019 ± 0.335
*L. rossiae* Fi40	1.122 ± 0.106	0.464 ± 0.098	4.364 ± 0.253
*L. rossiae* Fi21	1.268 ± 0.097	0.417 ± 0.069	3.110 ± 0.274
*L. brevis* Fi30	1.293 ± 0.089	0.368 ± 0.062	2.741 ± 0.290
